# Inertial Measuring System to Evaluate Gait Parameters and Dynamic Alignments for Lower-Limb Amputation Subjects

**DOI:** 10.3390/s24051519

**Published:** 2024-02-26

**Authors:** Shao-Li Han, Meng-Lin Cai, Min-Chun Pan

**Affiliations:** 1 Department of Mechanical Engineering, National Central University, Taoyuan 32001, Taiwan; 2 Department of Physical Medicine and Rehabilitation, Changhua Christian Hospital, Changhua 500209, Taiwan

**Keywords:** wireless inertial measuring system, Madgwick filtering, ZUPT, dynamic alignment, prosthetic gait analysis

## Abstract

The study aims to construct an inertial measuring system for the application of amputee subjects wearing a prosthesis. A new computation scheme to process inertial data by installing seven wireless inertial sensors on the lower limbs was implemented and validated by comparing it with an optical motion capture system. We applied this system to amputees to verify its performance for gait analysis. The gait parameters are evaluated to objectively assess the amputees’ prosthesis-wearing status. The Madgwick algorithm was used in the study to correct the angular velocity deviation using acceleration data and convert it to quaternion. Further, the zero-velocity update method was applied to reconstruct patients’ walking trajectories. The combination of computed walking trajectory with pelvic and lower limb joint motion enables sketching the details of motion via a stickman that helps visualize and animate the walk and gait of a test subject. Five participants with above-knee (*n* = 2) and below-knee (*n* = 3) amputations were recruited for gait analysis. Kinematic parameters were evaluated during a walking test to assess joint alignment and overall gait characteristics. Our findings support the feasibility of employing simple algorithms to achieve accurate and precise joint angle estimation and gait parameters based on wireless inertial sensor data.

## 1. Introduction

Gait deviation among subjects with lower limb amputation is unavoidable even with the latest powered prosthesis [1,2,3]. Unilateral amputation causes balance difficulties during walking [4], but gait deviations vary with time and training. Gait asymmetry after wearing a lower extremity prosthesis poses a lifelong health threat to amputees [5]. The altered biomechanism worsens gait asymmetry and increases the consequent development of lower back pain, osteoarthritis, and skin problems [6]. These problems result in transfemoral amputees relying heavily on their sound limbs during walking [3,7], and then worsening the overuse arthropathy. Despite these situations, a prosthesis helps subjects regain their socioeconomic functions after amputation [8]. Researchers proved that after receiving four weeks of endurance training, lower-limb amputees were able to reduce step widths, similar to healthy subjects [9], i.e., rehabilitation for amputees also changes the biomechanics after wearing a prosthesis [2,10]. Thus, assessing the quality of a prosthesis and monitoring the gait deviations are essential for these changes.

Fit and alignment have been always the concerns of wearing a prosthesis. To judge if the prosthesis fits is, however, a challenge. Until now, the appropriateness of a prosthesis is subjectively reported by amputees; meanwhile, how well-aligned a prosthesis is is always observed by a prosthetist [11]. The definition of alignment for a prosthesis user is the relative motion between the socket and the other parts of a prosthesis [12]. With good feasibility, the alignments after wearing a prosthesis have been always subjectively clarified by a prosthetist [11]. To handle this issue, the alignments, gait parameters, and the symmetry of gaits have been taken as objective variables to show a good fit for a prosthesis [11,12]. To obtain objective variables that represent fitness, the alignments of interest can be further divided into static and dynamic ones [12]. Static alignment after wearing a prosthesis can be obtained by connecting affixed makers on the amputee’s surfaces and prosthetics [8]. The method is intuitive and easy to find the discrepancy between the unaffected and amputated legs. However, the research clarifies that static alignment may not directly relate to ground reaction forces (GRF) due to the compensatory mechanisms adopted by amputees to adapt to alignment changes [13]. The limitations of static gait analysis and the compensatory mechanisms employed by amputees necessitate the development of novel, clinically applicable gait analysis tools, capable of dynamically assessing joint alignment during walking, to facilitate a comprehensive evaluation of gait parameters in post-prosthetic amputees.

Dynamic alignment refers to the assessment of the relative motion between prosthetic components and the individual’s body parts to optimize their functionality during walking. It involves the coordination of the socket, artificial knee joint, and artificial foot to ensure a smooth and efficient gait for the amputee. In contrast to the traditional static alignment that primarily emphasizes aligning the prosthesis while the user is stationary, dynamic alignment encompasses the complete three-dimensional joint ranges of motion during walking. Traditionally, dynamic alignments have been assessed by prosthetists through observing amputees’ walking patterns and listening to their feedback and concerns [12,13,14]. For decades, to acquire dynamic alignments, optical motion capture systems have been regarded as a gold standard. They can provide objective and reliable gait analysis for lower limb prostheses users [14]. Time-consumption and facility limitation hinder their clinical applications. As far as lower-extremity amputees are concerned, rehabilitation may improve gait deviation which varies over time after wearing a prosthesis [2,9]. Furthermore, a gait analysis performed in a controlled laboratory environment does not correlate well with functional results in daily activities [15]. Thus, those motion capture systems are somewhat not practically feasible for clinical use.

Implementing a wearable device system is one of the solutions to obtain dynamic alignments for amputees to overcome the challenges in continuously monitoring prosthesis fitness and training programs. Inertial sensors have played a critical role in wearable devices for gait analysis [5,16,17,18]. An inertial sensor comprises an all three-axis accelerometer, gyro, magnetometer, and other compact sensors. Acceleration during walking has been considered as an indicator of walking smoothness [19]. Further, the calculated trunk stability can be used as a surrogate scale for functional improvement among transfemoral amputees [9]. Thus, inertial sensors are suitable for amputees to monitor pelvic motion and gait symmetry, which is reasonable for comprehensive rehabilitation. Using inertial sensors alone to classify gait cycles through motion data is challenging [5]. Gait analysis for amputees is more complicated than for healthy subjects and the debate still remains for phase discrimination through using inertial sensors. Most previous studies have typically focused on subjects with either transfemoral or transtibial amputation [20,21,22]. Limited researchers have investigated both types of amputation in their studies [23]. To cope with this issue, we developed a comprehensive assessment tool that incorporates clinical walking tests. The tool provides prosthetics and rehabilitation specialists with an intuitive and readable means to evaluate patients.

Most studies have achieved promising results by using sophisticated algorithms to process motion data obtained from healthy subjects, but still few studies have directly obtained motion data from amputees [18,24,25]. The other crucial issue is to decide where to deploy inertial sensors. The proper locations to deploy inertial sensors on amputee subjects have not been conclusively determined. The fewer sensors the system has, the better the practicability for amputee use. An inertial sensor deployed on the back is suitable for embedding into back belts as decoration. The kinematics of the lower extremities such as the pelvic and trunk motions are crucial for gait analysis among amputees [9]. An inertial sensor affixed on a lateral socket prosthesis is also proper for transfemoral amputees. Generally, inertial sensors are deployed on the trunk and the upper portions of the legs, which are accessible sensors for amputees to without further bending their trunk to affix sensors on the lower limbs for motion analysis. This hypothesis should be further justified.

The study aims to conduct a dynamic alignment analysis, in comparison to the unaffected leg; further, both the large- and small-scale motion analyses, i.e., walking trajectory and joint-angle variation, are implemented and combined to illustrate subject movements through stickman animation. In addition to allowing clinical physicians to watch prosthetic users’ walking conditions, visualization can also allow prosthetic users to understand their gait patterns. Notably, viewing the walk-test animation together with the physician’s explanation may enhance the patient’s motivation to perform the rehabilitation task. Previous studies considered dynamic alignment as the positioning of the prosthesis concerning the residual stumps [26,27]; however, our primary concerns here are paid to the overall impact and net effect on gait analysis and, even in recent research, no suitable method has been available to monitor joint motion among amputees wearing prostheses [28]. Depicting dynamic alignment in this manner offers an intuitive approach for both amputees and prosthetists. The trend for gait analysis and dynamic alignments has still not been able to visualize gait variations [29,30,31].

## 2. Material and Methods

In this section, we describe the construction of the inertial measuring system and the methods to clarify the gait phase, rotational angles of each joint on the sagittal plane and spatiotemporal parameters and to draw the gait animation using the information of both the walking trajectory and the lower-limb joints motion, which benefit amputees and prosthetists to intuitively comprehend gait deviations. First, we set up the kinematic analysis system with seven inertial sensors. Then, the gold standard, a video-based motion capture system, was employed to validate this inertial measuring system for the obtained kinematic parameters and walking trajectory. Finally, we adopted this system on amputees to integrate with a clinical walk test. The study protocol for enrolling amputees was approved by the Landseed International Hospital Institutional Review Board (IRB) with the identification code IRB-20-022-A2. All participants gave signed informed consent before starting all tests.

### 2.1. Wireless Inertial Measuring System

**Figure 1:**
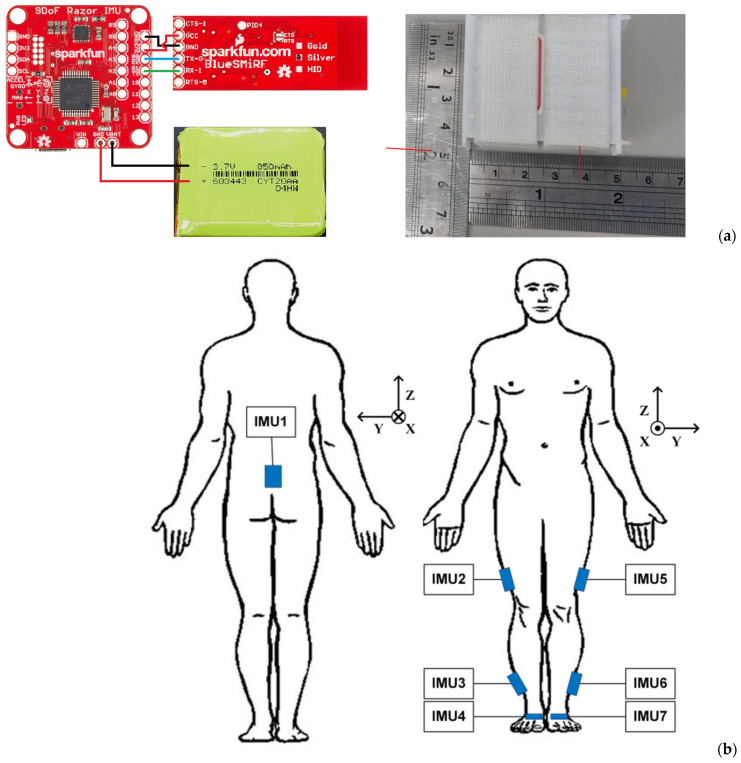
Wireless IMUs and their use: (**a**) composition of a single IMU, (**b**) illustration of the coordinate system and mounting locations of IMUs.

The implemented inertial system comprised seven wireless inertial measurement units (IMUs) and a laptop computer (ASUS X571G with 16 G RAM) and a USB port with a USB dongle (CSR Bluetooth 4.0) serving as the data storage center and receiving port. Each IMU contained an inertial module (Razor IMU M0 Module, SparkFun Electronics, Niwot, CO, USA), a Bluetooth^®^ module (Blue SMiRF Silver Bluetooth module, SparkFun Electronics, Niwot, CO, USA), and a LiPo battery with 3.7v 850 mAh, as shown in Figure 1a. The specification of the employed inertial module (Razor IMU M0 Module) included a three-axis accelerometer measuring (selectable) ±2, ±4, ±8, and ±16 g, a three-axis gyro measuring ±250, ±500, ±1000, and ±2000°/s, and a three-axis magnetometer with a measuring range of ±4800 μT. This module was selected as it allows for inhouse programming that provides flexibility in data acquisition. Before starting the study, we placed one IMU set on the dorsum of a healthy subject’s foot to examine the maximum acceleration and angular velocity during a 10 m walk test. The results showed that the acceleration and angular velocity ranged from −2.93 to 1.27 g and from −297.4 to 464°/s, respectively.

**Figure 2:**
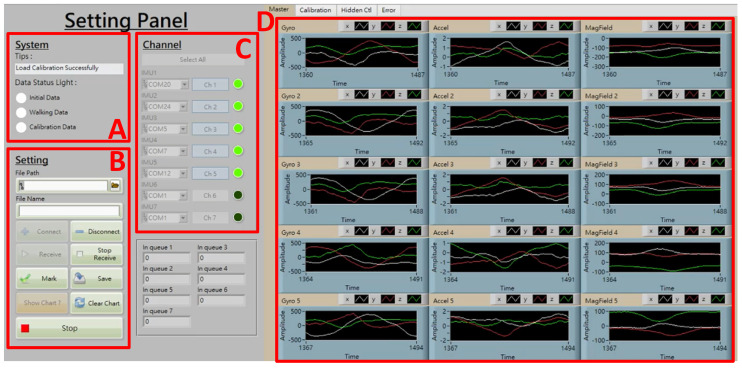
Operation interface to acquire inertial data, where section (**A**): status monitoring, (**B**): function keys, (**C**): IMU connection and data acquiring, (**D**): illustration of acquired inertial data (from left to right column for angular velocity, acceleration, and magnetic field, respectively).

The measuring dynamic range for the Gyro and accelerometer were set as ±500°/s and ±4 g, respectively, with a sampling rate of 100 Hz. The IMU was packed by polylactic acid, 51 × 46 × 24.5 mm in size, and weighed 38 g. Hook and loop fasteners were used to affix these sensors on subjects. As shown in Figure 1b, the mounting locations to deploy seven IMUs included the subjects’ mid-back (on the spinal process of the fifth lumbar vertebrae), both sides of the lateral thigh, lateral calf, and foot dorsum, etc. The walking direction was set toward the positive *X*-axis, and the direction of gravity as the negative *Z*-axis (Figure 1b). The operation interface to acquire inertial data of multiple IMUs was coded using LabVIEW^®^ (National Instruments, Austin, TX, USA), as shown in Figure 2, where one may find in section C that the light green bulbs represent Ch1-Ch5 IMUs in operation. Before performing a test to obtain motion data, users need to calibrate the gyro and accelerometer of each IMU.

### 2.2. Computation Algorithms

To describe the orientations of inertial sensors with respect to the global coordinate system, the Euler angles were used as the fundamental basis for the computation of kinematic parameters. To prevent the situation of gimbal-lock during the computation of rotation of the coordinate system, the quaternion [32] was applied for the expression of attitude orientation. Further, Madgwick filtering [33] and zero velocity update (ZUPT) [34] were adapted to correct accumulated errors from integral and drift in the inertial data computation.

#### 2.2.1. Rotation and Coordinate Transformation of Orientation

As orientations approaching gimbal-lock may introduce associated errors, the quaternions offer an alternative computational method. A rigid body or coordinate frame in a three-dimensional (3D) space can be represented by a four-dimensional complex number that is called quaternion [32]. When an arbitrary frame *A* rotates angle α around a vector r→A to frame *B*, the elements of quaternion describing this orientation are written as

(1) q^BA=[q0q1q2q3]T=[cosα2−rxsinα2−rysinα2−rzsinα2]T

where *r_x_*, *r_y_*, and *r_z_* are the components of r→A on the x, y, and z axis, respectively. The orientation described by quaternion can be represented as a rotation matrix RBA, i.e., [22,25]

(2) RBA=[2q02-1+2q122(q1q2+q0q3)2(q1q3-q0q2)2(q1q2-q0q3) 2q02-1+2q222(q2q3+q0q1)2(q1q3+q0q2)2(q2q3-q0q1)2q02-1+2q32]

#### 2.2.2. Madgwick Filtering for Quaternion Correction

The Madgwick algorithm is a recursive procedure as it uses the previous quaternion to estimate the current one. The adjustment and correction of quaternion of an IMU is obtained through the gradient descent by using its rotation axis and the information of measured linear and gravitational acceleration. Then, the angular displacement for roll, pitch, and yaw can be evaluated.

First, consider the estimation of orientation for the gyro in an IMU. The gyro measures the three angular velocities of the sensor frame, termed *ω_x_*, *ω_y_*, and *ω_z_*, related to its local (or sensor) coordinate system. These angular velocities compose a quaternion ωS with a null as the real part like

(3) ωS=[0ωxωyωz]

Thus, the orientation change from sensor frame to earth frame can be calculated by

(4) q˙ES=12q^ES⊗ωS

The orientation of the earth frame relative to the sensor frame in time *t*,  qω,tES can be computed by integrating the derivative of quaternion q˙ω,tES, i.e.,

(5) q˙w,tES=12q^est,t−1ES⊗ωtS,

(6) qw,tES=q^est,t−1ES+q˙ω,tESΔt.

If the measured gyro data are not null even though the subject stays idle, then the integration of angular velocity yields errors in angular displacement, which may accumulate along with time dramatically. The errors can be corrected by using Madgwick filtering. Consider the estimation of orientation for the accelerometer in the IMU. The accelerometer measures both the motion and gravity acceleration that can be used to correct errors resulting from gyro through the computation algorithm. The employed quaternions in the computation need a complete solution, and thus it regards optimization. Assume the quaternion of sensor to earth q^ES, the predefined reference direction of the field in the earth frame d^E, and the measured direction of the field in the sensor frame s^S, it expects the quaternion q^ES can be resolved as a solution to the optimization of

(7) minq^ES∈ℜ4f(q^ES,d^E,s^S),

where the objective function f(q^ES,d^E,s^S)=q^∗ES⊗d^E⊗q^ES−s^S, with the other terms including q^ES=[q1q2q3q4], d^E=[0dxdydz], and s^S=[0sxsysz]. To handle an optimization problem, the most frequently used approach is the gradient descent algorithm. It results in an estimate of the orientation q^nES for *n* iterations based on the orientation of the ‘initial guess’ q^0ES and a step-size µ [33]. To sum up, the Madgwick algorithm is based on angular velocity to obtain the optimal solution as a reference to correct gyro posture. After adopting this computation scheme, one can obtain accurate kinematic parameters from inertial sensing data.

#### 2.2.3. ZUPT for Gait Phase Detection

**Figure 3:**
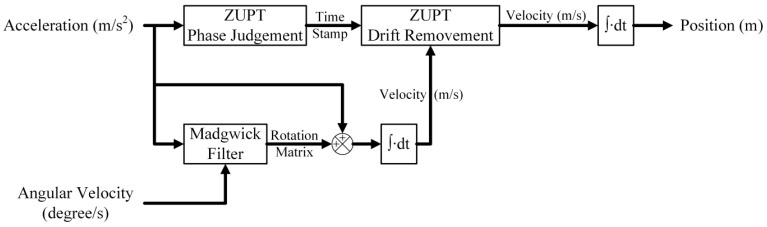
Flow diagram to reconstruct subjects’ walking trajectories using Madgwick filtering and ZUPT.

The computation scheme of ZUPT results from the idea applying the feature remaining in each gait of walking [34]. Within the study, the inertial navigation to reconstruct walking trajectory combines both the Madgwick algorithm and ZUPT, as shown in Figure 3.

**Figure 4:**
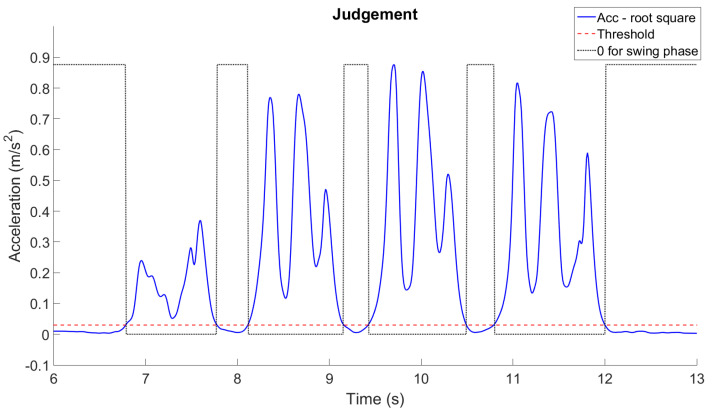
Setting of markers (dash line) for swing and stance phases through using acceleration data.

**Figure 5:**
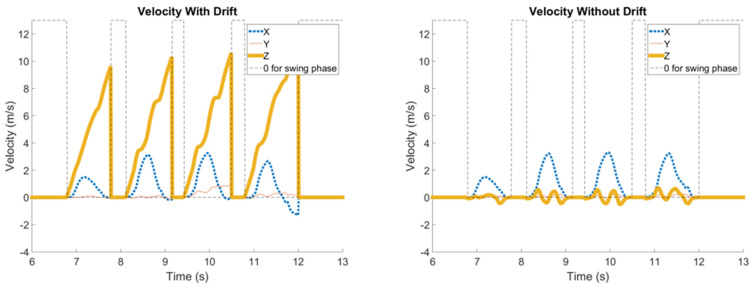
Adoption of the ZUPT scheme to correct drift errors.

The ZUPT contains two main parts. The first is to judge the gait phase. During the stance phase, the acceleration on the foot is supposed to reach the lowest level in each gait cycle. Therefore, we calculate the absolute value from the three axes of acceleration for IMU4 (Figure 1b) in each recorded time. A band filter ranging from 0.001 to 5 Hz was used to eliminate gravity and artifact noise. Then, the total acceleration was evaluated and used to set a marker for the stance phase by using acquired x-, y-, and z-direction acceleration of the IMU on the instep of the healthy side. As an example, Figure 4 illustrates the setting of the marker (dash line) to identify stance phases from gait cycles. The second is to reduce drift errors from the computation of inertial data. Since the stance instances are separated from gait cycles, the velocity of the foot can then be assigned as zero to reset drifts and calculated errors when evaluating displacement from the acceleration data, as illustrated in Figure 5. In order to obtain correct and accurate walking trajectory, recording the limb lengths of the test subject beforehand is essential. Though the ZUPT scheme has been proven to effectively identify varied phases in gaits while inertial sensors are employed, researchers have been still conducting additional research efforts to determine the stance phase for computation accuracy [35].

### 2.3. Kinematic Parameters and Gait Visualization

**Figure 6:**
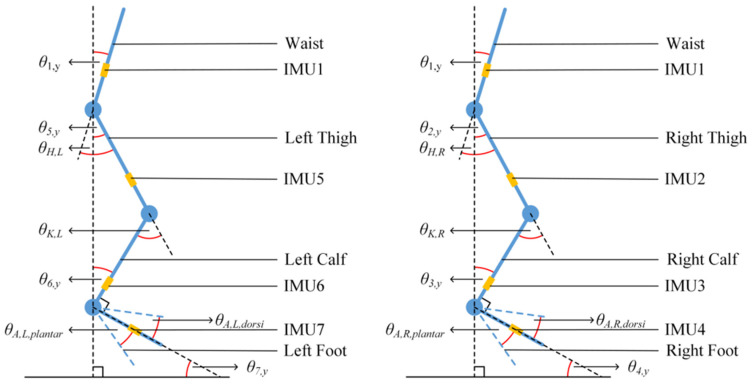
Definition of geometric relationship among the joints and trunk.

The range of motions (ROMs) for hip, knee, and ankle joints during walks are the rotational angles around the pitch. Figure 6 shows the definition of their geometric relationship for the six joints of lower limbs. The ROM of the pelvis can be obtained intuitively from the gyro of the IMU located on the back. The formulae to calculate each joint angle can be expressed as below, i.e.,

(8) $$\theta_{H,L}=\theta_{1,y}-\theta_{5,y},\theta_{H,R}=\theta_{1,y}-\theta_{2,y},$$

(9) $$\theta_{K,L}=-\theta_{5,y}+\theta_{6,y},\theta_{K,R}=-\theta_{2,y}+\theta_{3,y},$$

(10) $$\theta_{A,L}=\theta_{6,y}-\theta_{7,y},\theta_{A,R}=\theta_{3,y}-\theta_{4,y}.$$

where $\theta_{H,L}$/$\theta_{H,R}$, $\theta_{K,L}$/$\theta_{K,R},\;{and\;\theta }_{A,L}$/$\theta_{A,R}$ denote the ROM (θ) for the hip (*H*), knee (*K*), and ankle (*A*) joint; *R* and *L* represent the right and left extremity, respectively. Some other parameters such as the gait speed and average stride can be directly calculated through conducting a walk test at a given distance. The gait speed (*v*) in m/min is calculated by

(11) v=d/t,

where *d* and *t* represent the walk distance in *m*, and the recorded time duration is in min. Likewise, the average stride (*s*) in *m* can be calculated by

(12) s=v/c,

where *c* denotes cadence (stride/min). Note that *c =* 60 *f_h_*, with *f_h_* being the stride frequency in Hz. Obtaining an accurate stride is helpful, and the gait speed and cadence have been proven to correlate with functional status [36,37]. Within the study, the 10 m walk test in clinical settings was performed to evaluate those gait parameters. Such a test has been demonstrated to closely relate to subjects’ daily life ability [38]. Computing exact stride length from using inertial sensors is still challenging due to the drift and the integral errors [37,39,40]. As addressed above (Figure 3), both Madgwick filtering and ZUPT were applied to handle the issue.

#### Gait Visualization

**Figure 7:**
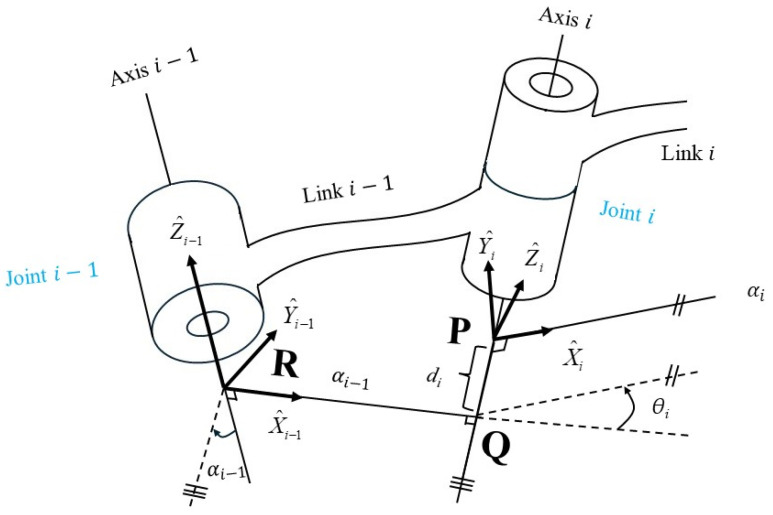
Definition of coordinate systems on two consecutive joints of robot links. (Redrawn based on [37] and the content in the article).

Combining the computed walking trajectory with the motion of the pelvic and lower limb joints enables one to sketch the details of motion via a stickman that helps visualize and animate the walk and gait of a test subject. One can consider the former (walking trajectory) and the latter (joint motion) as the macro and micro information of walking, respectively. To visualize joint motion by using the stickman, one needs to model lower limbs and derive the space positions of joints. Madgwick filtering and quaternion are combined to evaluate the Euler angles of joints (Figure 3); further, the Denavit–Hartenberg (D-H) parameters [41] were adopted to obtain instant joint positions. Here, the forward kinematics were applied to evaluate joint space positions through equating the lengths and calculated Euler angles of the links (limbs). For modeling the lower limbs for the pelvis, together with the left- and right-side limbs, first the local coordinate systems for all joints and links (limbs) need to be defined, that is ${\hat{X}}_{i-1}$-${\hat{Y}}_{i-1}$-${\hat{Z}}_{i-1}$ and ${\hat{X}}_{i}$-${\hat{Y}}_{i}$-${\hat{Z}}_{i}$ for Joint *i −* 1 and Joint *i* respectively, and Axis *i −* 1, Axis *i,* and Link *i −* 1 between Joint *i −* 1 and *i* (for *i* = 1 to *N*), as Figure 7 shows. Note that the $\hat{Z}$ axis coincides with joint axis, and the later ${\hat{Z}}_{i}$ should be vertical or parallel to ${\hat{Z}}_{i-1}$; ${\hat{X}}_{i-1}$ is a common vertical axis to ${\hat{Z}}_{i-1}$ and ${\hat{Z}}_{i}$, and the $\hat{Y}$ axis can be decided according to the right-hand rule. Following the above rule, then four D-H parameters can be decided, that is, (i) the link length *a_i−_*_1_ between ${\hat{Z}}_{i-1}$ and ${\hat{Z}}_{i}$, (ii) the twist angle *α_i−_*_1_ from ${\hat{Z}}_{i-1}$ rotating to ${\hat{Z}}_{i}$ around ${\hat{X}}_{i-1}$, (iii) the joint angle *θ_i_* from ${\hat{X}}_{i-1}$ rotating to ${\hat{X}}_{i}$ around ${\hat{Z}}_{i-1}$, and (iv) the link offset *d_i_* between ${\hat{X}}_{i-1}$ and ${\hat{X}}_{i}$. Figure 7 also defines five transformations of coordinate systems from {*i −* 1} to {*R*}, {*Q*}, {*P*}, and {*i*} sequentially. For each transformation, only one degree of freedom varies, and thus it employs one parameter. The procedure to transforming Joint *i* to Joint *i −* 1 comprises 4 steps including a rotation *α_i−_*_1_ from {*i −* 1} to {*R*}, a translation *a_i−_*_1_ from {*R*} to {*Q*}, a rotation *θ_i_* from {*Q*} to {*P*}, and eventually a translation *d_i_* from {*P*} to {*i*}. The combination of 4 transformations yields

(13) Pi−1=TRi−1TQRTPQTiPPi=Tii−1Pi,

where Tii−1 denotes the so-called the D-H transformation matrix, i.e.,

(14) Tii−1=RX(αi−1)DX(ai−1)RZ(θi)DZ(di)=[10000cαi−1−sαi−100sαi−1cαi−100001][100ai−1010000100001][cθi−sθi00sθicθi0000100001][10000100001di0001]=[cθi−sθi0ai−1sθicαi−1cθicαi−1−sαi−1−sαi−1disθisαi−1cθisαi−1cαi−1cαi−1di0001]

Note that *cθ_i_ = cosθ_i_* and *sθ_i_ = sinθ_i_.* Tii−1 can be substituted to evaluate each joint. Assuming the number of joints *N*, the transformation matrix, TN0=T10T21T32⋯TNN−1, can be expressed as

**Figure 8:**
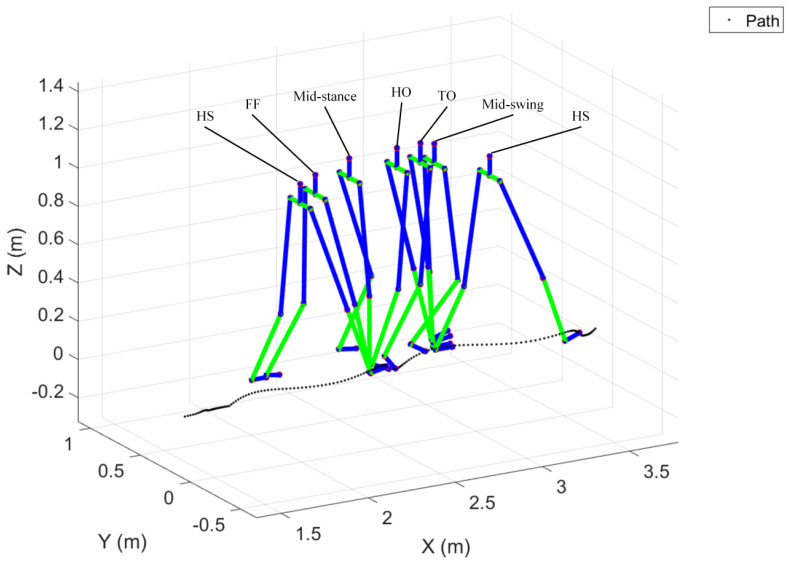
Reconstruction of walking trajectory. (The colors in the figure from top to down represent the pelvis, thigh, calf, and foot).

(15) TN0=[r11r12r13Px,Nr21r22r23Py,Nr31r32r33Pz,N0001],

where *r*_11_, *r*_12_, …, *r*_32_, *r*_33_ are the rotation factors, and *P_x,N_*, *P_y,N_*, and *P_z,N_* denote the position coordinates of Joint N relating to T0. Within the study, Equation (15) was used to evaluate the position coordinates of lower-limb joints. Thus, the instant positions of lower limbs can be demonstrated through a stickman animation. The reconstruction of joint motion along the pitch can be implemented through obtaining the locations of all lower-limb joints. Together with the walking trajectory, the macro and micro gaits can be visualized, and Figure 8 shows one of examples. This intuitive animation provides both subjects and prosthetists with a gait pattern to adjust and monitor the fitness of a prosthesis.

## 3. System Validation

To validate the inertial measuring system with computation schemes for gait variables and dynamic alignment, both a three-axes precision rotating platform (Tanlian^®^ E-O Co., Ltd., Taoyuan City, Taiwan) composed of three single rotary modules (MR-90G, MR-120G, MR-160G) and an IRB 120 robot (ABB^®^, Zürich, Switzerland) were employed to examine the reliability and validity of the developed inertial measuring system [42]. Further, a commercialized optical motion capture system (Qualisys^®^ Motion System Oqus 700+, Göteborg, Sweden) was employed as the gold standard for comparison.

(1) ABB^®^ IRB 120 robotics

**Figure 9:**
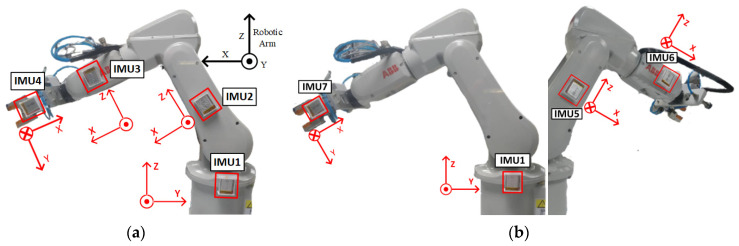
(**a**,**b**) illustrate the simulation of the right and left lower limbs, respectively, and show the setup of IMUs to validate the measuring system through using ABB^®^ IRB 120 robot, and illustration of the definition of local coordinate systems. The numbering of IMUs is the same as in Figure 1, which is used to simulate the mounting positions of IMUs on the lower limbs.

**Figure 10:**
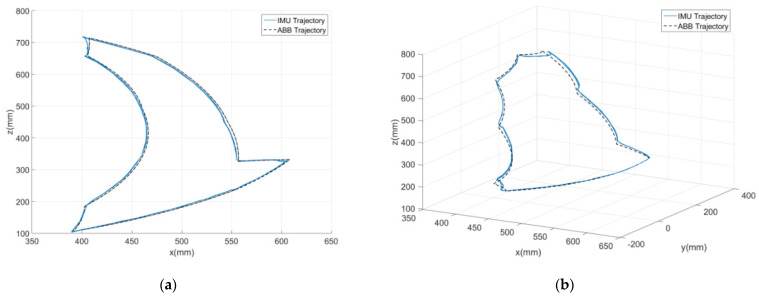
Robot motion trajectories (designated and IMU reconstructed) for (**a**) 2D and (**b**) 3D motion tasks.

The motion trajectories of the robot end-effector using IMU data were reconstructed and compared with the designated trajectories followed by assigned rotation on the robot joints. The designated motion trajectories provided by an industrial robot like ABB^®^ IRB 120 used here can be considered an even more reliable standard and comparison basis than an optical motion capture system since all the trajectory data are generated by a machine and computation code. Figure 9 shows the setup of seven mounted IMUs on the robot to be validated. One IMU was mounted on one robot arm for the reconstruction of motion trajectories using inertial data. But, it is noted that we designated IMU1, 2, 3, and 4, and IMU1, 5, 6, and 7 to mimic their installation on the two lower limbs. Two types of motion tasks (2D and 3D) were designed to compare the reconstructed trajectories for the end-effector between using inertial data and robot designated paths, as shown in Figure 10. One was a two-dimensional (2D) plane motion that mimics an adult’s gait in a walk. The other was a three-dimensional (3D) motion to further consider the inclusion of hip joint movement. Seven valid data sets (*N* = 7) were acquired to evaluate the root mean squared error (RMSE) and error percentage (EP) for each motion task, i.e.,

(16) $$RMSE=\sqrt{\frac{1}{N}\sum_{i=1}^{N}\left[{\left(x_{i}-x_{i,ideal}\right)}^{2}+{\left(y_{i}-y_{i,ideal}\right)}^{2}+{\left(z_{i}-z_{i,ideal}\right)}^{2}\right]},\mathrm{and}$$

(17) $$EP=\sqrt{\frac{\sum_{i=1}^{N}\left[{\left(x_{i}-x_{i,ideal}\right)}^{2}+{\left(y_{i}-y_{i,ideal}\right)}^{2}+{\left(z_{i}-z_{i,ideal}\right)}^{2}\right]}{\left[{\left(x_{ideal,range}\right)}^{2}+{\left(y_{ideal,range}\right)}^{2}+{\left(z_{ideal,range}\right)}^{2}\right]}}\times 100\%,$$

where *N* is the total number of IMU measured points, (*x_i_*, *y_i_*, *z_i_*) and (*x_i_*_,*ideal*_, *y_i_*_,*ideal*_, *z_i_*_,*ideal*_) denote the reconstructed coordinates using IMU data and the designated coordinates of the robot end-effector, and (*x_ideal_*_,*range*_, *y_ideal_*_,*range*_, *z_ideal_*_,*range*_) characterize the motion range of the end-effector in the *x*, *y,* and *z* directions. As shown in Table 1, the RMSEs and EPs are within 6 and 1 mm, respectively, for the 2D motion task with a total path length of 5209 mm and 15 and 2 mm for the 3D motion with a total path length 8307.3 mm. Table 1 compares the computed motion trajectories using inertial data with the designated ABB^®^ IRB 120 robot, showing good results and illustrating relative accuracy on the inertial measuring system. In the 2D motion tasks, the RMSE and EP remain at the largest at 5.94 ± 1.31 mm and 0.92 ± 0.2%; in the 3D motion tasks, the RMSE and EP are at the largest at 14.56 ± 1.12 mm and 1.78 ± 0.14%, respectively.

(2) Qualisys^®^ Motion System

**Figure 11:**
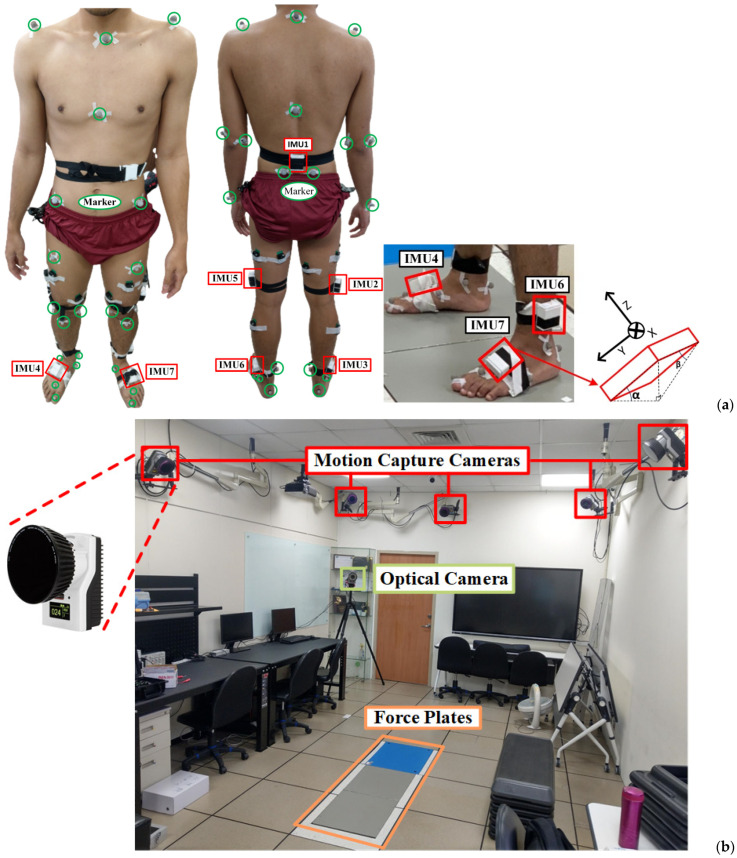
Validation of the developed inertial measuring system: (**a**) subject wearing IMUs and optical markers, (**b**) setup of optical motion capture system (Qualisys^®^), where the force plates are the 1.8 m marked region. The yellow box represents the positions of the force plates.

**Figure 12:**
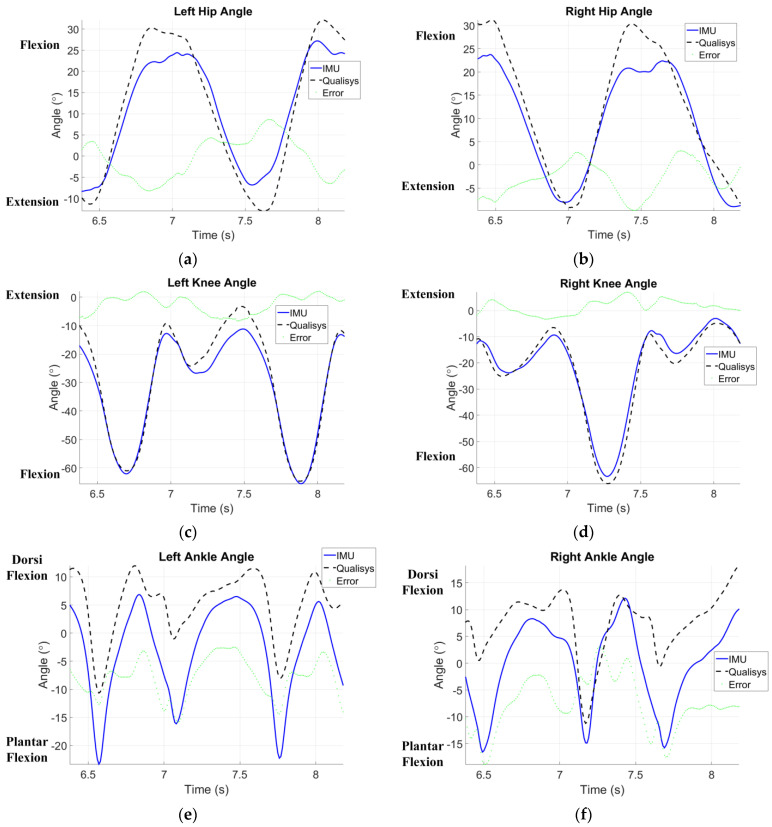
Comparison of evaluated extension/flexion of (**a**,**b**) hip, (**c**,**d**) knee, and (**e**,**f**) ankle joint between the results from inertial data and Qualisys^®^. The depicted figures demonstrate the effectiveness of the proposed algorithms in aligning the measurements of the inertial sensors with those of the high-speed camera system.

To validate experimentally the inertial measuring system against an optical motion, a tracking system was conducted on healthy subjects. It was considered that evaluating the accuracy of the system on healthy subjects can convey equivalent confidence for the use by healthy or amputee subjects. Conducting a head-to-head comparison between the inertial measuring system and optical motion capture system, especially on amputation subjects, is a time-consuming task due to the variability in the placement of markers and sensors. The suitable locations for optical markers and inertial sensors obviously vary among lower limb amputees with different levels of amputation. Further, the complexity and difficulty of the test process on the amputation subjects may raise the IRB review issue. Basically, this comparison needs a bigger number of subjects, longer distances to perform and repeated tests. As shown in Figure 11a, the optical markers and seven IMUs were affixed to test subjects to perform a 4.2 m walk, composed of 1.2 m to start, 1.8 m marked recording (region of three force plates in Figure 11b), and 1.2 m to end, for the gait analysis. In the optical system, eight motion capture cameras acquire marker images and one more video records motion pictures. We employed adhesive tape (3M^TM^) and hook and loop straps for fixing the IMUs. In the stage of the amputee patients’ walking test, only the hook and loop straps for fixation were used. Before each comparison test, 2 sec stationary data were acquired from subjects standing still on the start line to initiate sensor orientation and to align with optical markers. All evaluated Euler angles using the initial IMU data were reset and calibrated to zero. Within the study, six healthy adults were recruited for the validation, and five valid tests per subject were recorded and analyzed. The correlation coefficient (*CC*) was evaluated as below [42]

(18) $$CC=\frac{\sigma_{\theta_{IMU}}{\;}_{\theta_{Video}}}{\sigma_{\theta_{IMU}}\sigma_{\theta_{Video}}}=\frac{\sum_{n=1}^{N}\left(\theta_{IMU}(n)\theta_{Video}(n))-N({\bar{\theta }}_{IMU}{\bar{\theta }}_{Video}\right)}{\sigma_{\theta_{IMU}}\sigma_{\theta_{Video}}}$$

to compare the waveform similarity, where ${\sigma_{\theta_{IMU}\theta }}_{Video}$ means the covariance of calculated joint flexion/extension from the inertial measuring system and the optical motion capture system, $\sigma_{\theta }$ denotes the standard deviation, $\theta_{IMU}(n)$ and $\theta_{Video}(n)$ are the calculated joint flexion/extension at the time instant *n*, ${\bar{\theta }}_{IMU}$ and ${\bar{\theta }}_{Video}$ are the mean values, and N is the data length. The larger the *CC* value (close to 1), the more resembled means. Figure 12 shows a comparison of evaluated extension/flexion of hip, knee, and ankle joints for one of the subjects between the results from inertial data and Qualisys^®^ with *CC* values of 0.97, 0.98, and 0.85, respectively [42]. Table 2 summarizes an overall comparison for individual lower-limb joints. Note that the RMSEs for hip and knee joint angles are both less than 7°, the *CC* values are up to 0.96, and the EPs are in between 12 and 21%. The revealed discrepancy of the results between the inertial data and Qualisys^®^ arises partly from the mounted IMUs inclined to the back, limbs, and insteps; especially, the inclined IMUs on the insteps vary along with individual subjects (Figure 11a). Compared with the hip and knee joints, a larger discrepancy for the ankle joint arises from IMU inclination to the instep. The compensation and correction to handle initial IMU inclination should be considered. Additionally, the above-mentioned discrepancy of joint angles partially comes from the computation of the optical motion capture system (Qualisys^®^). Table 1 compares the computed motion trajectories using IMU data with the designated one of the ABB^®^ IRB 120 robot. It is noted that the result from the optical motion capture system is a relative standard, but errors may still result from the measuring, data-handling, and computing stages.

## 4. Clinical Application on Amputee Subjects

**Figure 13:**
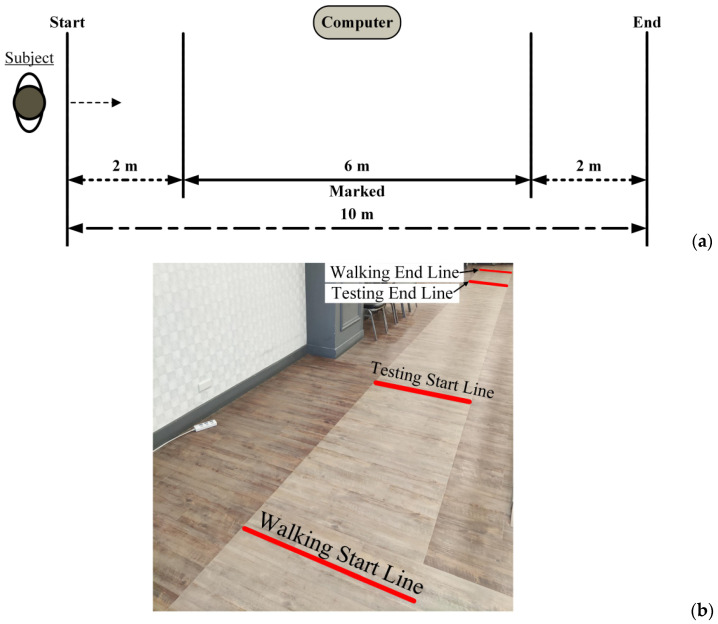
Route of 10 m walking test, (**a**) schematic and (**b**) photo.

**Figure 14:**
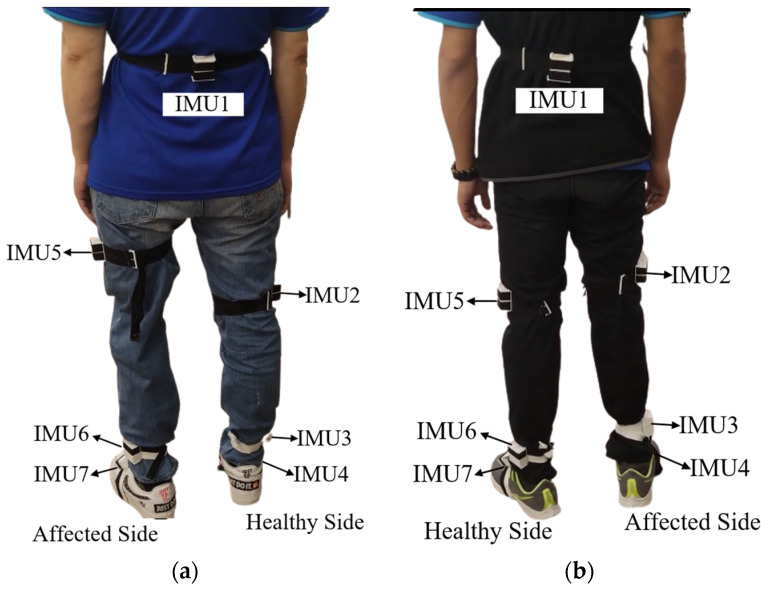
IMUs mounted on prosthesis-wearing amputee participants, (**a**) transfemoral and (**b**) transtibial.

Due to the heterogenicity of amputee-specific assessment scales, a clinical 10-m walk test, as shown in Figure 13, is more suitable and feasible to obtain the average walking speed [43], one of critical variables to assess the functional status for amputees with equipped prosthesis [9]. For the feasibility test, we recruited amputees who were capable of walking independently after wearing a prosthesis. Participants must be alert and oriented, and capable of walking without assistance to complete 10-m walk test at once. To exclude other obscure comorbidities, only subjects who lost their lower limbs because of trauma were enrolled. Furthermore, to obtain steady gait patterns, amputee participants have been using a prosthesis to walk for over three years. Five amputee adults, right-handed, were enrolled, as shown in Table 3 giving their demographic data. Additionally, one healthy adult (right-handed, aged 25 years, 175 cm tall, and 95 kg weight) also completed the test as a reference. After wearing their prostheses, all subjects walked on flat terrain for 10 m at a comfortable speed. While the inertial sensors were affixed, IMU 2, 3, 5, and 6 may be deployed at different levels because of the absence of limbs. Figure 14 shows the sites to deploy sensors for the transfemoral and transtibial amputee.

**Figure 15:**
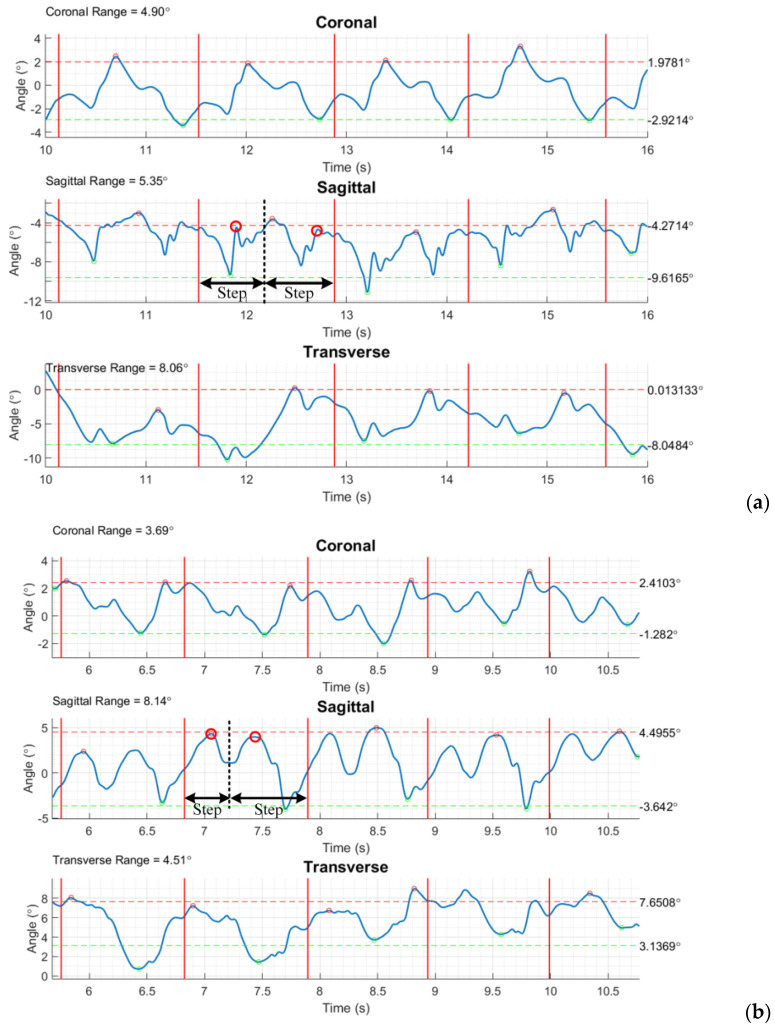
Pelvis rotation on three planes for (**a**) healthy and (**b**) amputee subject (#1), where the ROMs on the individual planes are characterized at the up-left corner. (Each two consecutive vertical solid lines ‘―’ characterize a stride. The rotation on the sagittal plane is able to show two steps in a stride, where dash vertical lines ‘- - -’ divide the steps, and circles ‘o’ indicate the instants of the left- and right-thigh farthest rotating position in the step.)

**Figure 16:**
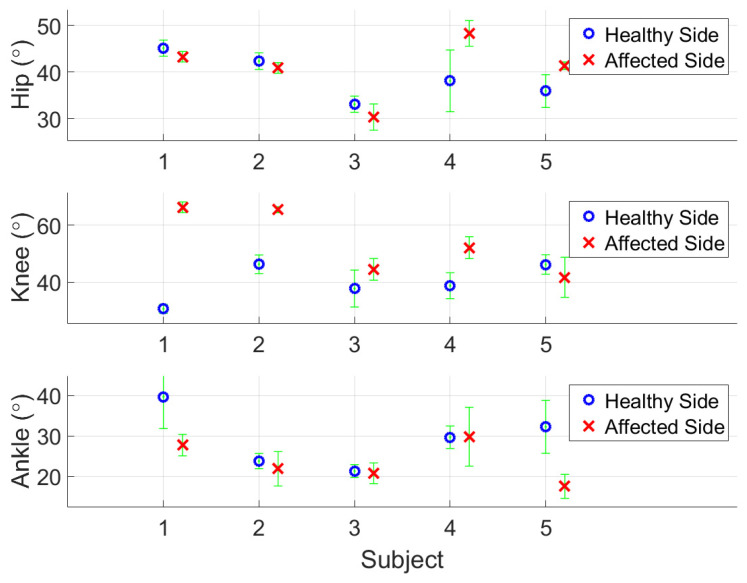
Comparison of ROMs for hip, knee, and ankle joints (from top to bottom) along the sagittal plane between sound and prosthetic legs.

Figure 15 illustrates pelvic rotation of test subjects (healthy and above-knee amputee) on three planes as examples. Table 4 gives the calculated gait parameters, including the average walking speed, cadence, step length, lower back (pelvis) rotation on three planes, and the ROMs of lower-limb joints for both healthy and prosthetic legs. To perform a dynamic alignment analysis, Figure 16 illustrates the assessment of the relative motion between the prosthetic components and the individual’s body parts, where the ROMs of joints are compared in pairs for the affected and healthy sides. One may also refer to Table 3 showing the demographic data of enrolled amputee participants. The results exhibit that the ROMs of the affected-side knee are apparently large for transfemoral amputation (Subject #1 and #2), the ROMs of the affected-side knee are a bit large, and the ROMs of the affected-side ankle are relatively small for transtibial amputation (Subject #3, #4 and #5). It is noted that more recruited subjects are still needed to confirm the above results.

In the calculation, the ranges of pelvis rotation on individual planes can be characterized from the considered gait cycles. The feasibility test demonstrates the availability of kinematic parameters, including gait phase discrimination, pelvic rotation (tilt and obliquity) and lateral displacement, and the aforementioned reconstruction of walking trajectory with lower-limb joints motion. All subjects have greater ROMs in affected knee joints. According to the previous study, increased ROMs in affected knee joints provide more stability for amputees [44]. All enrolled subjects can walk without any walking device and the results support their conclusion. However, the disagreement exists in other variables. The ROMs in hips and ankles can be influenced by the various artificial knee and ankle joints among our subjects. Therefore, it is inappropriate to make a clarified conclusion.

## 5. Discussion

*Justified inertial measuring system*. Using a three-axes rotating platform and ABB^®^ robotics to validate the inertial measuring system, in Table 2 the ROMs show very good correlation with the optical motion tracking system, Qualisys^®^, with *CC* values of 0.96 and 0.98 for the hip and knee, respectively, if the whole gait cycles were considered. When the swing and stance phases were chosen to compare separately, the correlation with the results from Qualisys^®^ still shows high agreement in the ROMs of the hip and knee joints (in Table 2, the *CC* range for hip ROMs is 0.88–0.93 and for knee ROMs is 0.83–0.97). As regards the *CC* values for ankle ROMs, the range is 0.79–0.94. The deployed IMU inclined to the foot, as illustrated in Figure 11a, needs compensation for the measured inertial data. Compensation will be applied using orthogonal transformations to correct the measurement errors caused by the inclination of IMU installation. The compensation for sensor mounting inclination can be achieved by utilizing the properties of orthogonal transformation to correct the orientation of the coordinate axes in case of any inclination. The computation employs the Z-Y-X Euler Angle Rotation Matrix, RBA, composed of *R_x_R_y_R_z_* to rotate the sensor coordinate directions to be consistent with the global coordinate system. Additionally, the impact at the moment of heel contact may exacerbate drift errors of inertial data; thus, an additional correcting scheme should be considered for a long walk distance.

*Computation efficiency and affordability*. Our system demonstrates high accuracy in measuring joint angles during short-distance walking, as evidenced by the strong correlation (0.81 ± 0.08 to 0.98 ± 0.01) with high-speed camera measurements (Table 2 and Figure 12). It shows potential for a reliable and affordable alternative to traditional motion capture systems, especially considering the significant cost efficiency of the seven sensors used. Beyond the evaluation of joint angles, the system can capture additional gait variables such as phase discrimination, walking speeds, and trunk motion. This comprehensive data collection further enhances its value for gait analysis. However, further research is still needed to explore its applicability in long-distance walking and long-term monitoring scenarios.

*Best mounting locations of IMUs*. The ROMs of hip and knee joints during walking can be individually evaluated by two IMUs beside (above and below) the joint. Moreover, the gait phases can also be differentiated by the calculated ROMs. Huong et al. concluded that three inertial sensors (acquiring acceleration and angular velocity) is the minimum requirement to distinguish various gait phases well [24]. Our results supported their viewpoints. Three IMUs, one on the back and two on the lateral thighs, are essential to gain satisfactory results. While using more sensors generally leads to a more comprehensive analysis, finding the optimal placement for IMUs on lower limb amputees is not a one-size-fits-all solution. The complexity of compensated gait patterns among individuals with prostheses necessitates tailoring sensor placement to suit the specific goals of each study.

*Visualization of walk trajectory with lower-limb joints motion*. The detection of gait phases through processing inertial data by the ZUPT scheme has been proven reliable, as can also be seen in [35], showcasing that attaching an inertial sensor on the shoe is suitable for the purpose. Within the study, the evaluation of joint posture and motion is based on quaternion and Madgwick filtering. Forward kinematics using the D-H parameters were applied to visualize lower-limb walk trajectory with a stickman animation, as shown in Figure 13. This visual feedback provides accessible insights for both amputees and prosthetists. Amputees can observe their own gait patterns, identify areas for improvement, and monitor progress during gait training. Prosthetists can leverage these data to personalize gait training programs and optimize prosthetic design based on individual needs, potentially leading to improved gait efficiency and reduced injury risk.

Furthermore, the D-H parameters used in this study have similarities to constrained control methods for predicting and evaluating joint angles [45]. The complex motion pattern of human joints involves the interaction of muscles, bone structures, and tendons, which are all interrelated. Therefore, it is not surprising that D-H parameters and constrained control methods can both effectively measure joint motion angles. D-H parameters are more direct and intuitive, while constrained control methods are more consistent with the motion pattern of human joints.

*Individual or average stride.* Ahmed and Diaz succeeded to calculate accurate step length through inertial data [37]. Precisely estimating step length for individual steps can be used to characterize gait asymmetry. But the argument remains that subjects likely achieve varied tasks in daily work with different step lengths; thus, the existing step discrepancy may only mean specific gait pattern. Within the study, individual step length was not considered, but the average one, i.e., the stride Equation (15), was, which represents a kind of stable gait pattern (Table 4). Research studies [46,47,48] have indicated that relying solely on gait asymmetry assessment is insufficient when evaluating amputees. The gait symmetry is often evaluated as the ratio of spatiotemporal variables such as step length during the swing or stance phase. Ongoing debates have regarded the definition of gait symmetry. Functional assessment is the main concern within our study; thus, the gait symmetry for (left/right) step length and duration were not emphasized, but these parameters can also be evaluated by using our system. Further, the walking speed significantly relates to the functional status [48]. When the walking speed increases, the asymmetry decreases. It implies that gait asymmetry alone cannot serve as a reliable indicator for inferring functional status in amputees [49]. It is enough for clinical reference to administrate appropriate rehabilitation [40]. The result from a clinical walk test is straightforward and perceptible for clinical experts. Additionally, using IMUs in a walk test can bridge the gap between clinical assessment and wearable sensors. The spatiotemporal parameters that were evaluated and extracted from inertial data measured during a walk test correlate well with existing functional assessment tools [50].

*Joint alignment*. From Table 4, the transfemoral amputation subjects (subject 1 and 2) revealed less variability than those with transtibial amputation (subject 3–5) in alignments. Zahedi et al. concluded that the loss of knee control among subjects with transfemoral amputations contributed to the lower amount of variability [12]. Within the study the inertial sensors on the thigh, leg, and foot provide more motion parameters, which is consistent with their findings.

## 6. Concluding Remarks

Within the study, the implemented inertial measuring system enables the evaluation of reliable kinematic parameters for the subjects’ pelvic, bilateral lower-limb joints, gait features, and even animation of walk trajectory with joint motion from a regular walk test. These attributes indicate its feasibility and lower clinical application barriers. However, the fewer enrolled subjects limited the expansibility of the study results. An in-depth study should recruit more participants and include various daily activities, such as stair climbing, walking across obstacles, etc. Improper and careless fixation may result in sensor data drift. But there exists a trade-off to not impact the convenience of the wearability for the subjects. In our test circumstances, the walking distance and speed were not too long or too fast, so the drift problem caused by fixation can be ignored. Further, we performed sensor calibration for the IMUs before each measurement. As mentioned in (2) of Section 3, “System Validation”, to handle the discrepancy of the computed joint angles between the inertial data and Qualisys^®^, compensation and correction to the IMU-inclined orientation should be considered. It is worth noting that the result from the optical motion capture system is also a relative standard, i.e., the errors may still come from the measuring, data-handling, and computing stages. To sum up, we conducted a comprehensive study by enrolling both healthy subjects and amputees with trans-tibial or trans-femoral amputation to address a notable gap in the previous studies.

## Figures and Tables

**Table 1:** Comparison of the motion trajectories between the reconstruction by IMU sets (Left: IMU1, 2, 3, and 4; Right: IMU5, 6, and 7) and robot designation (# of test: 7), where the Left and Right IMU sets were mounted on two sides of the robot arm for validation, as shown in Figure 9; the latter is on the right- and left-hand-side limbs, respectively, for subject test.

Motion Tasks	RMSE (mm)	EP (%)	Designated Path Length (mm)
2D (Right)	5.94 ± 1.31	0.92 ± 0.2	5209
2D (Left)	4.51 ± 0.64	0.7 ± 0.1	
3D (Right)	13.49 ± 1.83	1.65 ± 0.22	8307.3
3D (Left)	14.56 ± 1.12	1.78 ± 0.14	

**Table 2:** Comparison of joint angles measured and evaluated by the inertial measuring system and optical motion capture system.

	Hip				Knee				Ankle			
	Left		Right		Left		Right		Left		Right	
RMSE (mm) (°) *	6.4 ± 0.62		6 ± 0.86		6.74 ± 1.71		6.16 ± 1.62		7.31 ± 1.54		8.22 ± 1.37	
*CC*	0.96 ± 0.01		0.96 ± 0.02		0.98 ± 0.01		0.98 ± 0.01		0.89 ± 0.04		0.81 ± 0.08	
EP (%)	20 ± 3.80		21 ± 5.30		12 ± 1.70		15 ± 8.10		37 ± 13.20		33 ± 12.10	
	Swing	Stance	Swing	Stance	Swing	Stance	Swing	Stance	Swing	Stance	Swing	Stance
RMSE (°)	7.21	6.46	8.2	6.74	7.45	5.4	8.42	6.06	8.88	4.26	8.99	6.17
*CC*	0.91	0.93	0.88	0.91	0.97	0.83	0.93	0.9	0.87	0.94	0.79	0.83

* RMSE: root mean square error, *CC*: correlation coefficient, EP: error percentage (*n* = 30, 6 healthy subjects with 5 valid tests).

**Table 3:** Demographic data of enrolled amputee participants *.

Subject #	Gender	Age (yr)	Weight (kg)	Height (cm)	Affected Side	Level of Amputation	Duration of Prosthesis Usage	Prosthesis
1	F	53	53	157	R	T/F	More than 20 years	Knee: Total Knee^®^ 2000
								Foot: FEEDOM Seirra^®^
2	M	43	75	178	L	T/F	More than 25 years	Knee: Total Knee^®^ 2000
								Foot: ottobock-1D35
3	M	26	60	172	R	T/T	More than 8 years	Foot: Pro-Flex^®^ XC
4	M	53	72	170	L	T/T	More than 40 years	Foot: Pro-Flex^®^ XC
5	M	45	95	178	L	T/T	More than 20 years	Foot: Pro-Flex^®^ XC

* Abbreviations: F for female and M for male; R for right and L for left; T/F and T/T stand for transfemoral and transtibial amputation, respectively. Readers may find more detailed specifications of the commercial prosthetic knees and feet from the producers’ homepages.

**Table 4:** Kinematic parameters evaluated by the inertial measuring system.

#	Speed (m/min)	Cadence (Step/min)	Stride (m)	Low Back Motion (°) *			ROMs of Healthy Legs (°)			ROMs of Prosthetic Legs (°)		
				C	S	T	Hip	Knee	Ankle	Hip	Knee	Ankle
1	73.9 ± 1.8	123.2 ± 3	1.2± 0	3.3 ± 0.4	7.9 ± 0.4	5.8 ± 1.5	45.1 ± 1.7	30.7 ± 1.6	39.7 ± 7.9	43.3 ± 1.1	66.2 ± 1.9	27.7 ± 2.6
2	67.6 ± 1.1	90.1 ± 1.5	1.5± 0	10.5 ± 0.4	13.4 ± 0.8	11.9 ± 0.5	42.3 ± 1.8	46.3 ± 3.3	23.8 ± 1.9	40.9 ± 1.1	65.4 ± 1.0	21.9 ± 4.2
3	70.6 ± 6.5	117.7 ± 10.9	1.2 ± 0	2.5 ± 0.8	3.9 ± 0.6	3.7 ± 0.6	33.1 ± 1.7	37.8 ± 6.5	21.4 ± 1.6	30.3 ± 2.8	44.5 ± 3.8	20.8 ± 2.6
4	78.1 ± 5.7	104.1 ± 7.6	1.5 ± 0	2.8 ± 0.3	3.8 ± 0.7	2.3 ± 2	38.1 ± 6.6	38.8 ± 4.5	29.7 ± 2.8	48.3 ± 2.8	52.1 ± 3.8	29.8 ± 7.3
5	78.3 ± 4.9	112.4 ± 5.8	1.4 ± 0.1	5.4 ± 0.6	11.8 ± 1.3	10 ± 1.3	35.9 ± 3.5	46.2 ± 3.4	32.3 ± 6.5	41.3 ± 0.8	41.8 ± 7.0	17.6 ± 2.9

* C: coronal, S: sagittal, T: transverse.

## Data Availability

Data underlying the results presented in this paper are not publicly available but may be obtained from the authors upon reasonable request.
